# Does wealth hinder structural coordination? Fiscal capacity and the coordination of healthcare and older adult care in China

**DOI:** 10.3389/fpubh.2026.1892675

**Published:** 2026-07-29

**Authors:** Hanhua Wang, Shijie Lin, Yingying Ji

**Affiliations:** 1 College of Traditional Chinese Medicine, Zhejiang Pharmaceutical University,Ningbo, China; 2 The First People's Hospital of Longwan District, Wenzhou, China

**Keywords:** China, county-level analysis, coupling coordination degree, older adult care beds, fiscal constraints, healthcare resources, population aging

## Abstract

**Objective:**

While population aging intensifies the need for integrated health and older adult care, it remains unclear whether greater local fiscal capacity actually facilitates such coordination. This study examines the coupling coordination between healthcare resources and older adult care beds at the county level in Ningbo, Taizhou, and Wenzhou (Zhejiang, China) from 2022 to 2024, with a focus on the role of fiscal constraints.

**Methods:**

A panel of 31 county-level units (*N* = 93) was constructed. A coupling coordination degree (CCD) model was used to measure the structural coordination between the healthcare subsystem (beds, physicians, and registered nurses per 1,000 registered population) and the older adult care subsystem (older adult care beds per 1,000 registered population). Factors associated with CCD were examined using a two-way fixed effects panel regression with county-clustered robust standard errors.

**Results:**

The mean CCD was 0.419 (SD = 0.166), indicating a state between “near disorder” and “barely coordinated,” with no significant temporal trend. The aging rate was positively associated with CCD (*β* = 0.128, *p* = 0.027). Counterintuitively, fiscal self-sufficiency exhibited a significant negative within-county association (*β* = −0.00181, *p* = 0.014): periods of higher own-source revenue reliance within a county were accompanied by lower coordination. GDP per capita and urbanization rate were not statistically significant. Robustness checks confirmed these findings.

**Conclusion:**

The negative within-county association between fiscal self-sufficiency and CCD reveals a “fiscal paradox”: periods of higher fiscal autonomy are accompanied by a relative under-prioritization of older adult care beds, creating a structural imbalance, whereas greater reliance on conditional transfers is associated with more balanced resource allocation. These findings generate the hypothesis—meriting further testing with direct transfer data—that conditional intergovernmental transfers may help promote balanced resource allocation, and that coupling indicators embedded in transfer designs could warrant evaluation in future policy pilots.

## 1 Introduction

Population aging is compelling governments worldwide to integrate healthcare and long-term care systems (1, 2). A widely held—yet rarely tested—assumption is that greater local fiscal capacity naturally facilitates this integration (3, 4). This study challenges that assumption by presenting within-county evidence from coastal China that reveals a striking paradox: periods of higher fiscal self-sufficiency are associated with declining structural coordination between medical and older adult care resources. To unpack this paradox, we situate our inquiry at the intersection of two bodies of literature.

The first body concerns the measurement of inter-system coordination, where the coupling coordination degree (CCD) model has emerged as a prominent tool (5). Originating in physics and later extended to social-ecological systems, CCD quantifies the extent to which two subsystems evolve in a mutually reinforcing manner (6). In health services research, this approach has been applied primarily at national and provincial scales to assess the alignment between health workforce supply and population health demand (7). A parallel stream of work has applied coupling models to examine the spatial matching between urban aging and older adult care facilities at the prefectural level (36). A handful of studies have extended the framework to the health–older adult care nexus in China, demonstrating its utility for capturing system imbalances (5, 8). However, these analyses remain overwhelmingly provincial or prefectural in scope (5). County-level evidence is strikingly absent—a critical gap, given that resource allocation, budget execution, and service delivery are operationalized at this administrative level in China. Without disaggregated analysis, cross-county heterogeneity within prefectures remains invisible, and the drivers of coordination at the frontline of service delivery go unexamined.

The second body of literature addresses fiscal capacity and the structure of public spending (9)—and it is here that our core theoretical tension lies. Classical fiscal federalism theory posits that decentralized governments with greater own-source revenues are better positioned to align spending with local preferences (10, 11). Applied to our context, this implies that wealthier counties should exhibit superior medical–older adult care coordination, as they possess both the means and the autonomy to respond to local demographic needs. However, a competing perspective—rooted in the political economy of intergovernmental transfers and bureaucratic incentives—suggests the opposite (12). Studies of local government spending behavior in China have documented a systematic bias toward “high-visibility” infrastructure projects at the expense of “low-visibility” social services, a pattern shaped by career incentives that reward tangible, large-scale achievements over routine welfare provision (12, 13). When own-source revenues are abundant and unconstrained by conditional transfer requirements, county governments may channel disproportionate investment into high-profile medical infrastructure—tertiary hospitals, advanced equipment, specialist recruitment—while neglecting the less politically rewarding domain of older adult care beds. By contrast, counties reliant on earmarked intergovernmental transfers face binding conditions that mandate simultaneous investment in both sectors. If this mechanism holds, we would observe a negative relationship between fiscal self-sufficiency and coupling coordination—a finding that would challenge the conventional assumption that fiscal abundance automatically promotes structural coordination between healthcare and older adult care resources.

Building on these two literatures, we formulate three hypotheses that guide our empirical analysis. First, the demand-pressure hypothesis (H1) posits that a higher proportion of older adults residents compels local governments to prioritize integrated care, generating a positive association between the aging rate and CCD (7). Second, the fiscal-autonomy hypothesis (H2)—our core theoretical contribution—posits that within-county increases in fiscal self-sufficiency are associated with lower CCD, because unconstrained fiscal discretion enables biased investment toward high-visibility medical projects at the expense of older adult care beds (9). Third, the institutional-conditionality hypothesis (H3) posits that counties heavily dependent on intergovernmental transfers achieve higher CCD, because transfer conditions require simultaneous allocation to both subsystems (14). This hypothesis draws on a well-established body of fiscal federalism scholarship demonstrating that earmarked intergovernmental transfers can correct vertical and horizontal fiscal imbalances and reshape subnational spending composition toward policy priorities that would otherwise be underfunded (11, 15). In the Chinese context specifically, recent evidence indicates that conditional transfers from central and provincial governments substantially restructure county-level expenditure patterns, shifting resources toward social services including healthcare and older adult care (3, 14). If this mechanism operates in our study area, we would expect counties with lower fiscal self-sufficiency—and therefore greater reliance on conditional transfers—to exhibit more balanced resource allocation between the two subsystems, not despite their fiscal constraints but because of the institutional conditions attached to the transfers they receive.

In this study, “integrated care” refers to the policy objective, while our empirical measurement captures the structural coordination of resource stocks between the two subsystems—specifically, the quantitative balance of beds, physicians, nurses, and older adult care beds—and does not encompass the processual dimensions of integrated care such as care coordination, multidisciplinary collaboration, or information sharing.

## 2 Methods

### 2.1 Study area and design

This study employed an explanatory sequential mixed-methods design (16), integrating a quantitative panel analysis of 31 county-level units with qualitative semi-structured interviews. The quantitative phase identified statistical associations, and the qualitative phase was conducted afterward to illuminate the mechanisms underlying these patterns. The study area comprised all counties, county-level cities, and districts within Ningbo, Taizhou, and Wenzhou—three prefectural-level cities in southeastern Zhejiang Province, China. This coastal region was selected for its substantial intra-regional heterogeneity in both economic development (GDP per capita ranging from approximately 50,000 to over 300,000 RMB) and fiscal capacity, providing natural variation to examine how local financial conditions shape the coordination of health and older adult care resources. The three cities have been active pilot sites for integrated medical–older adult care policies and long-term care insurance reforms, enhancing the policy relevance of our findings (17).

### 2.2 Quantitative data and variables

#### 2.2.1 Data sources and panel construction

A balanced three-year panel (2022–2024, *N* = 93) was constructed from official statistical yearbooks of Ningbo, Taizhou, and Wenzhou. Data on health institution beds, licensed (assistant) physicians, and registered nurses were extracted from the health chapters of each yearbook. Demographic and economic indicators—registered population, GDP per capita, budget revenues, and budget expenditures—were obtained from the population and economy chapters of the same yearbooks. Older adult care bed counts were sourced from the civil affairs chapters of the municipal statistical yearbooks. The urbanization rate and the aging rate (proportion of population aged 60 years and above) were obtained from the annual Main Data Bulletin on Population released by each municipal government. County-specific aging data were unavailable; thus, municipal averages were assigned to constituent counties. The implications of this proxy are addressed in Section 4.3.

#### 2.2.2 Variable definitions

The healthcare subsystem was characterized by three indicators, each calculated per 1,000 registered population: health institution beds, licensed (assistant) physicians, and registered nurses. The older adult care subsystem was measured by older adult care beds per 1,000 registered population. The dependent variable, CCD, was derived from the two subsystems (Section 2.3). Independent variables included aging rate (%, aged 60+), GDP per capita (log-transformed), fiscal self-sufficiency rate (local budget revenue / local budget expenditure × 100), and urbanization rate (%). Registered population served as the denominator for all per capita health and older adult care indicators, consistent with China’s resource planning framework. Spatial patterns of CCD were visualized using county-level choropleth maps with a natural breaks (Jenks) classification method (35).

### 2.3 CCD model

The CCD was calculated in three steps following established protocols (5, 18).

Step 1–Normalization. All indicators were min–max normalized across the full panel (93 observations) to eliminate differences in units and scale:

$$X_{\mathrm{ij}}=\frac{x_{\mathrm{ij}}-\min(x_{j})}{\max(x_{j})-\min(x_{j})}$$

where $x_{\mathrm{ij}}$ is the original value of indicator j for county-year i . Because normalization is performed across all 93 county-year observations, the minimum value of each indicator becomes 0 after transformation. Hence, a CCD of 0 indicates that the county-year observation had the minimum sample value for at least one subsystem indicator, not that the resource count itself was zero.

Step 2–Subsystem indices. The healthcare subsystem index $U_{1}$ was computed as the equally weighted average of the three normalized indicators (beds, licensed physicians, and registered nurses per 1,000 registered population). Equal weights were adopted for transparency and replicability. The older adult care subsystem index $U_{2}$ was the normalized value of older adult care beds per 1,000 registered population.

Step 3–Coupling degree (C), coordination index (T), and CCD (D).

$$C=\frac{2\sqrt{U_{1}\times U_{2}}}{U_{1}+U_{2}},T=\frac{U_{1}+U_{2}}{2},D=\sqrt{C\times T}$$

The CCD ranges from 0 (complete disorder) to 1 (optimal coordination). For descriptive purposes, CCD values were classified using a conventional ten-level scale; continuous values were retained for regression analysis. As a sensitivity check, we recalculated CCD using entropy-based weights (Supplementary Table S6).

### 2.4 Panel regression model

To identify factors associated with CCD, we estimated a two-way fixed effects model:

$$\begin{array}{l} CCD_{\mathrm{it}}=\beta_{0}+\beta_{1}\text{Agin}g_{\mathrm{it}}+\beta_{2}\ln(\text{GDPp}c_{\mathrm{it}})\\ +\beta_{3}\text{Fisca}l_{\mathrm{it}}+\beta_{4}\text{Urba}n_{\mathrm{it}}+\mu_{i}+\lambda_{t}+\varepsilon_{\mathrm{it}} \end{array}$$

where $\mu_{i}$ denotes county fixed effects (absorbing time-invariant heterogeneity such as geography and administrative culture), $\lambda_{t}$ denotes year fixed effects (capturing common temporal shocks, e.g., national policy changes), and $\varepsilon_{\mathrm{it}}$ is the idiosyncratic error. Standard errors were clustered at the county level to accommodate arbitrary within-county serial correlation. Model selection was guided by a Hausman test (comparing fixed vs. random effects) and an F-test of the joint significance of year dummies. Robustness checks included: (1) re-estimation excluding an outlier observation (CCD = 0); (2) alternative standard error specifications; and (3) sensitivity analysis using entropy-weighted CCD (Supplementary Table S6). All quantitative analyses were performed in R version 4.6.0 using the plm, lmtest, and sandwich packages.

Given the two-way fixed effects specification, all coefficients are identified from within-county variation over time and should be interpreted accordingly. We do not make cross-sectional claims about differences between counties. Standard errors are clustered at the county level (31 clusters) to accommodate arbitrary within-county serial correlation. The sandwich package’s default finite-sample correction (HC1) is applied. We note that the modest number of clusters and the short panel period (*T* = 3) warrant caution in interpreting *p*-values; results are best viewed as indicative associations rather than precise estimates.

### 2.5 Qualitative interviews

To illuminate the mechanisms underlying the quantitative associations—particularly the counterintuitive fiscal finding—we conducted semi-structured interviews with key informants in six purposefully selected counties (two from each prefectural city). Selection followed a maximum-variation logic across two dimensions: fiscal self-sufficiency (high vs. low) and aging rate (high vs. moderate). A total of 12 informants were recruited, including professionals with direct involvement in resource planning and allocation from county health bureaus (*n* = 4), civil affairs bureaus (*n* = 4), and finance departments (*n* = 4). All informants held positions that required direct engagement with budget formulation or service planning for medical or older adult care during the study period.

Interviews were conducted in person between Feb and Apr 2026, after the quantitative panel period (2022–2024). The accounts obtained are therefore retrospective with respect to the budget allocation processes under study and should be interpreted as contextual evidence that illuminates plausible mechanisms, rather than as contemporaneous observations. To mitigate recall bias, interview questions focused on general patterns and recurring priorities in budget formulation (e.g., “In a typical budget cycle, which types of projects tended to receive priority?”) rather than on specific numerical figures or isolated events. Interviews were conducted at the informants’ workplaces (*n* = 9) or in neutral settings such as meeting rooms in municipal government service centers (*n* = 3), according to informant preference. Each interview lasted 40–70 min (mean = 52 min). All interviews were conducted by the first author, who has postgraduate training in qualitative methods and prior experience conducting interviews with public sector professionals in China. A semi-structured topic guide was developed based on the three hypotheses (H1–H3) and covered: (1) the annual budget formulation process for health and older adult care; (2) perceived priorities and trade-offs in resource allocation; (3) the influence of intergovernmental transfers and their associated conditions; and (4) the local implementation of national integrated medical-older adult care policies. The guide was piloted with two informants not included in the final sample, and minor wording adjustments were made to improve clarity.

Transcripts were analyzed using thematic analysis (19), combining deductive and inductive approaches. An initial coding framework was deductively derived from the three hypotheses (H1: aging-driven demand pressure; H2: fiscal autonomy and expenditure bias; H3: transfer conditionality and balanced allocation). Two members of the research team (the first author and a co-author) independently coded the first four transcripts (one from each prefectural city and one additional) using this framework. The coders then met to compare their coding, resolve discrepancies through discussion, and refine the coding framework by adding emergent inductive codes (e.g., “bureaucratic inertia,” “landmark vs. routine spending”). The first author then coded the remaining eight transcripts using the refined framework, with the co-author independently coding a random sample of three of these transcripts to confirm consistency (inter-coder agreement > 90%). Emergent themes were identified through iterative reading and cross-case comparison. Data saturation was assessed throughout the analysis; after coding ten transcripts, no substantially new themes emerged, and the final two transcripts confirmed thematic saturation. All coding was performed manually without qualitative data analysis software.

The qualitative component was designed not to test the three hypotheses independently, but to provide contextual depth for interpreting the panel regression results—specifically, to elucidate the mechanisms through which fiscal self-sufficiency may shape the coordination of medical and older adult care resources. The qualitative findings are therefore positioned as hypothesis-generating and interpretive, rather than as independent verification of the quantitative results. The COREQ checklist, interview topic guide, and coding framework are provided in Supplementary File S7.

### 2.6 Integration of quantitative and qualitative findings

Integration occurred primarily at the interpretation stage. Quantitative results from the panel regression were first used to establish statistical patterns. Qualitative themes were then mapped onto these patterns to elucidate the mechanisms driving the observed associations, particularly the negative relationship between fiscal self-sufficiency and CCD. This explanatory sequential logic—quantitative patterns informing qualitative inquiry, with qualitative findings enriching quantitative interpretation—follows established mixed-methods practice (20, 21).

## 3 Results

### 3.1 Descriptive statistics

Table 1 reports the descriptive statistics of all variables over the study period (2022–2024). The mean CCD was 0.419 (SD = 0.166), with a minimum of 0.000 and a maximum of 0.703. The average aging rate (population aged 60 years and above) was 25.0% (SD = 3.1%), ranging from 20.6 to 30.2%. Considerable variation was observed in fiscal self-sufficiency (mean = 62.5%, SD = 24.9%, min = 20.1%, max = 116.1%), urbanization rate (mean = 71.8%, SD = 14.2%), and GDP per capita (mean = 121,001 RMB, SD = 57,673 RMB). These disparities across counties justify the panel regression analysis. Descriptive statistics disaggregated by year are presented in Supplementary Table S1.

**Table 1:** Descriptive statistics of all variables (pooled sample, 2022–2024, *N* = 93).

Variable	Mean	SD	Min	Max
Beds per 1,000 registered population (beds)	6.74	4.02	1.82	20.20
Physicians per 1,000	4.89	2.40	2.34	11.44
Nurses per 1,000	5.10	2.94	1.98	13.57
Older adult care beds per 1,000	5.01	3.09	1.65	18.96
GDP per capita (RMB)	121,001	57672.63	49,171	337,602
Fiscal self-sufficiency rate (%)	62.46	24.92	20.14	116.14
Aging rate (%, aged 60+)	24.97	3.14	20.58	30.15
Urbanization rate (%)	71.82	14.19	46.12	99.92
CCD	0.419	0.166	0.000	0.703

GDP per capita is presented in original units (RMB yuan). CCD = 0.000 occurred for one county-year observation (Yongjia, 2024). Under min–max normalization across the full panel, the county with the minimum value of older adult care beds per 1,000 registered population receives a normalized score of U₂ = 0, which mechanically produces CCD = 0. This does not imply a literal absence of older adult care beds.

**Figure 1:**
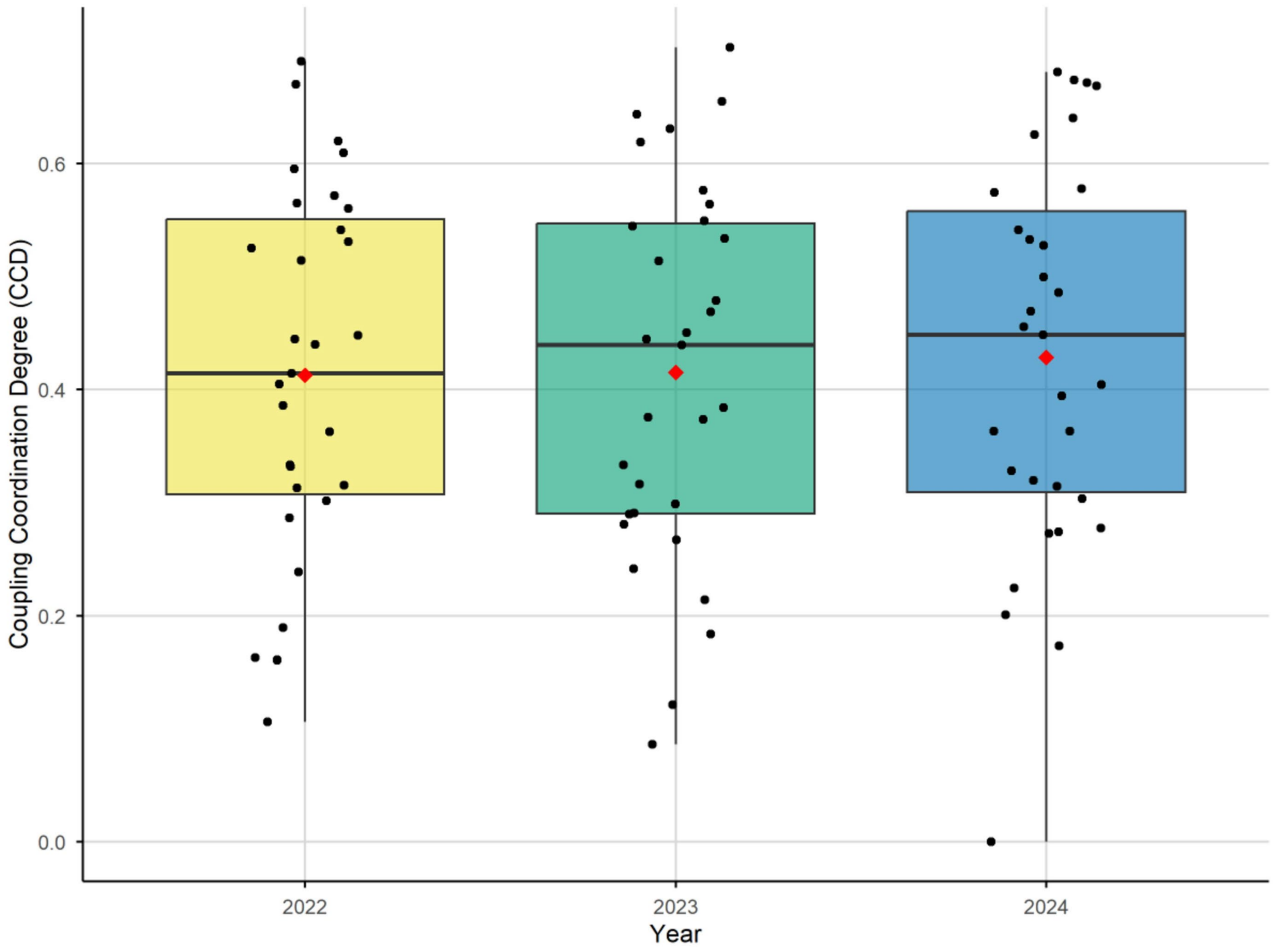
Boxplots of CCD by year, 2022–2024. The boxes represent the interquartile range (IQR), the middle line is the median, the red diamonds indicate the mean, and the black dots show individual county-year observations.

As shown in Figure 1, the CCD exhibited modest variation across the study period. The median CCD rose marginally from 0.430 in 2022 to 0.435 in 2024, but the interquartile ranges overlapped considerably, suggesting no statistically significant trend over the 3 years.

**Figure 2:**
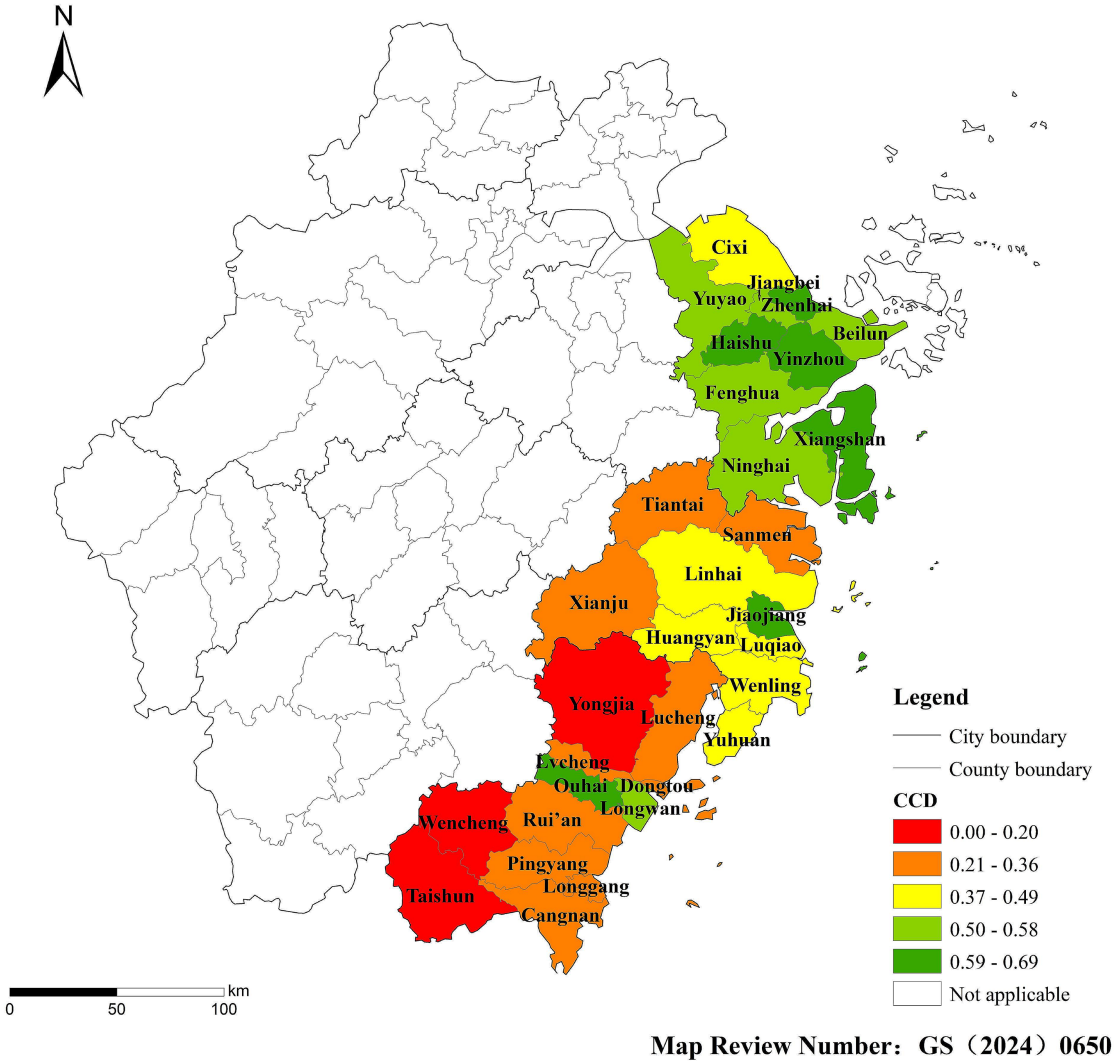
Spatial distribution of CCD in the coastal region of Ningbo, Taizhou, and Wenzhou, Zhejiang province, 2024. Counties are shaded using a natural breaks (Jenks) classification on a red-to-green diverging gradient, with red indicating lower CCD and green indicating higher CCD. Classification breaks are displayed in the legend. The map reveals pronounced spatial heterogeneity: Urban districts in northern and central areas generally exhibit higher coordination, whereas mountainous and peripheral counties—Particularly in southern Taizhou—fall into the lowest coordination categories. Notably, the spatial distribution is consistent with the within-county negative fiscal–CCD association reported in the regression analysis: Fiscal self-sufficiency and structural coordination do not co-locate spatially.

The boxplots in Figure 1 suggest overall stability over time but mask the pronounced cross-county disparities. Figure 2 maps the spatial distribution of CCD for the most recent year (2024) and reveals a stark geographic pattern: urban districts in northern and central areas generally exhibit higher coordination, whereas mountainous and peripheral counties—particularly in southern Taizhou—fall into the lowest coordination categories. Notably, counties with high fiscal self-sufficiency do not cluster in the high-CCD category, which visually anticipates the negative fiscal–CCD association confirmed in the regression analysis below.

### 3.2 Panel regression results

Table 2 presents the results of the two-way fixed effects model. The model is statistically significant overall (*F*(4,56) = 3.02, *p* = 0.025). Consistent with the demand-pressure hypothesis (H1), periods with higher aging rates within a county are positively associated with CCD (*β* = 0.128, *p* = 0.027), indicating that intensifying demographic pressure is accompanied by more coordinated resource allocation over time.

**Table 2:** Panel regression results (dependent variable: CCD).

Variable	Coefficient (cluster-robust SE)
Aging rate (%)	0.128* (0.0565)
Log GDP per capita	0.180 (0.1708)
Fiscal self-sufficiency rate (%)	−0.00181* (0.000714)
Urbanization rate (%)	0.000086 (0.000613)
Observations	93
*R^2^* (within)	0.177
Adjusted *R^2^*	−0.351
Model F-statistic (df = 4,56)	3.02*

Standard errors clustered at the county level are shown in parentheses. The model includes county and year fixed effects. Negative adjusted *R*-squared results from the large number of fixed effects relative to the short panel period. *p < 0.05.

The most striking and counterintuitive result concerns fiscal capacity. Within counties, fiscal self-sufficiency exhibits a significant negative association with CCD (*β* = −0.00181, *p* = 0.014). In substantive terms, a within-county increase of one percentage point in the fiscal self-sufficiency rate is associated with a 0.00181 decrease in CCD. This finding directly contradicts the conventional expectation that growing fiscal autonomy would lead to improved coordination; instead, the opposite pattern is observed.

GDP per capita (log-transformed) and the urbanization rate do not attain statistical significance in this specification (*p* = 0.296 and *p* = 0.889, respectively), suggesting that within this relatively prosperous coastal region, coordination is shaped more by institutional and incentive structures than by aggregate economic output or urban concentration. The within-group *R*^2^ of 0.177 indicates that the model explains a modest share of the within-county variation in CCD over time. The negative adjusted *R*^2^ (−0.351) reflects the large number of fixed effects relative to the short panel period (*T* = 3, *N* = 93) and underscores that the model is best interpreted as identifying associations observed within a short panel rather than providing a comprehensive explanation of CCD dynamics.

### 3.3 Robustness checks

We conducted several robustness checks to validate the main findings.

First, we re-estimated the model with heteroskedasticity and autocorrelation-consistent (cluster-robust) standard errors, as reported in Table 2. The significance levels of aging and fiscal self-sufficiency remained unchanged, confirming that the results are not driven by within-county error correlation.

Second, we examined the sensitivity to an extreme value by excluding the county-year observation with CCD = 0.000 (Yongjia, 2024). As shown in Supplementary Table S2, the coefficients of aging rate (*β* = 0.090, *p* = 0.035) and fiscal self-sufficiency (*β* = −0.00144, *p* = 0.026) remained statistically significant, although their magnitudes slightly decreased. The overall model fit was acceptable (*R*^2^ = 0.129), indicating that the main results are not driven by a single outlier.

Third, a Hausman test comparing fixed effects and random effects models strongly rejected the random effects specification (*χ*^2^(4) = 21.03, *p* < 0.001), favouring the fixed effects approach.

Fourth, we formally tested the joint significance of the year fixed effects using an F-test. The null hypothesis that both year dummies are zero was rejected (*F*(2,56) = 3.66, *p* = 0.032), confirming that controlling for common time trends is necessary. Moreover, a model with only county fixed effects (excluding year effects) failed to detect a significant effect of aging (*β* = 0.006, *p* = 0.355), underscoring the importance of accounting for temporal shocks (e.g., national policies on integrated medical-older adult care).

Fifth, we examined the sensitivity of our findings to alternative panel estimators. Table S3 reports the results from pooled OLS, random-effects, and two-way fixed effects specifications. The coefficient on fiscal self-sufficiency was negative and statistically significant only in the fixed-effects model (*β* = −0.0018, *p* < 0.05), whereas it was non-significant in pooled OLS (*β* = 0.0009, *p* > 0.1) and random-effects (*β* = −0.0010, *p* > 0.1) specifications. This pattern confirms that our central finding is contingent on controlling for unobserved county-level heterogeneity: without accounting for time-invariant county characteristics, the fiscal paradox remains concealed. The divergence across estimators further reinforces the necessity of the fixed-effects approach, consistent with the Hausman test reported above.

Taken together, these robustness checks support the validity and stability of our main findings.

To further investigate the mechanism underlying the negative fiscal–CCD association, we decomposed CCD into its constituent subsystem scores and re-estimated the models using *U₁*, *U₂*, and their gap as dependent variables (Table 3).

**Table 3:** Decomposition analysis: subsystem scores and resource gap (two-way fixed effects).

Variable	*U*₁ (Healthcare)	*U*₂ (Older adult care)	Gap (*U*₁ − *U*₂)
Aging rate	0.0465 (0.0367)	0.1305* (0.0771)	−0.0841 (0.0807)
Log GDP per capita	0.0595 (0.1292)	0.6224 (0.6888)	−0.5629 (0.6533)
Fiscal self-sufficiency	0.0000 (0.0005)	−0.0015* (0.0008)	0.0015 (0.0010)
Urbanization rate	−0.0004 (0.0003)	0.0009 (0.0011)	−0.0013 (0.0013)
Observations	93	93	93
*R^2^* (within)	0.018	0.140	0.058
Adjusted *R^2^*	−0.345	−0.189	−0.295
County FE	Yes	Yes	Yes
Year FE	Yes	Yes	Yes

Cluster-robust standard errors (county level) in parentheses. U₁ = equally weighted average of normalized beds, physicians, and nurses per 1,000 registered population; U₂ = normalized older adult care beds per 1,000 registered population. Gap = U₁ − U₂. *p < 0.1.

## 4 Discussion

### 4.1 Principal empirical findings

This mixed-methods study yields three principal findings that, taken together, challenge conventional assumptions about the relationship between fiscal capacity and structural coordination between healthcare and older adult care resources.

First, the overall coupling coordination between healthcare resources and older adult care beds across the 31 counties remained low (mean CCD = 0.419), with no statistically significant improvement over the 2022–2024 period. This finding extends prior national-scale assessments (5, 7) but with a crucial added insight: even within a relatively prosperous coastal region, substantial variation in structural coordination exists across counties and over time. The fixed-effects specification reveals that these dynamics are not merely cross-sectional differences but reflect within-county changes. The spatial heterogeneity we document—visualized in Figure 2—cannot be explained by aggregate economic development alone; more targeted institutional explanations are required.

Second, the aging rate was positively and significantly associated with CCD (*β* = 0.128, *p* = 0.027), supporting the demand-pressure hypothesis (H1). This finding aligns with the broader literature on demographic drivers of service integration (5, 7), but adds a policy-relevant nuance: demographic pressure does not mechanically produce integration. Rather, as our qualitative data indicate, this pressure operates through a threshold effect. Officials in counties with moderate aging rates described a bureaucratic inertia in which fragmented departmental responsibilities remained unchallenged until citizen complaints and unmet needs reached a critical mass. One health bureau official characterized this shift succinctly: “After the aging rate crossed roughly 23 percent, integrated care went from being a policy slogan on documents to a performance target that actually shaped our budget requests.” This threshold dynamic suggests that the positive coefficient of aging captures not a gradual adjustment but a punctuated response to escalating demand.

Third, the within-county negative association between fiscal self-sufficiency and CCD (*β* = −0.0018, *p* = 0.014) is driven primarily by a relative contraction of older adult care resources rather than by an expansion of healthcare resources. The decomposition analysis (Table 3) clarifies this pattern: fiscal self-sufficiency is unrelated to the healthcare subsystem score (*U*₁, *β* ≈ 0, *p* = 0.99) but is marginally and negatively associated with the older adult care subsystem score (*U*₂, *β* = −0.0015, *p* = 0.065). The coefficient on the resource gap (*U*₁ − *U*₂) is positive (*β* = 0.0015) but imprecisely estimated (*p* = 0.14), a pattern directionally consistent with a widening structural imbalance that the current panel has limited power to resolve at conventional significance levels.

This finding refines, rather than invalidates, the fiscal paradox interpretation. Our qualitative interviews indicate that fiscally autonomous counties favoured landmark medical projects in budget discussions, yet this preference did not translate into a measurable within-county expansion of the overall healthcare subsystem during the 2022–2024 study period. Instead, older adult care beds were treated as a residual category and, in some counties, declined relative to registered population. The net effect is a lopsided resource structure that emerges not because medical investment surges when fiscal autonomy increases, but because older adult care investment lags behind—a dynamic we label “uncoordinated fiscal autonomy.” The quantitative decomposition, combined with informants’ accounts that special-purpose transfers require bundled investment in both subsystems, suggests that conditional intergovernmental transfers may operate by protecting older adult care investment from residualisation, rather than by restraining medical overspending.

The non-significance of GDP per capita (log-transformed) and urbanization rate (*p* = 0.296 and *p* = 0.889, respectively) warrants brief comment. These null results suggest that, above the economic threshold represented by our coastal Zhejiang sample, further increases in aggregate affluence or urban concentration do not automatically translate into improved medical–older adult care coordination. Institutional design and incentive structures, rather than sheer wealth, are the binding constraints.

### 4.2 Theoretical interpretation and contributions

The empirical pattern documented above—periods of higher fiscal autonomy being associated with declining structural coordination—constitutes what we term the “fiscal paradox of structural coordination”: the condition under which fiscal abundance, rather than enabling balanced service provision, produces lopsided investment patterns that undermine cross-sectoral coordination. To explain this paradox, we propose the concept of “uncoordinated fiscal autonomy” —the dynamic in which local governments possess sufficient own-source revenues to pursue spending preferences shaped by political visibility rather than demographic need.

This concept engages, and in several respects challenges, three established bodies of literature.

First, our findings complicate a core tenet of classical fiscal federalism theory—that decentralized fiscal capacity improves the alignment between public spending and local preferences (15, 22). This proposition rests on an implicit assumption that local governments, when equipped with own-source revenues, will allocate resources in a manner that reflects the demographic needs of their constituents. Our results suggest a boundary condition: when career incentives reward visibility over balance, fiscal autonomy does not facilitate need-responsive allocation; instead, it enables the pursuit of politically salient investments at the expense of less conspicuous but equally vital services. This does not falsify fiscal federalism theory wholesale, but it indicates that its predictions are contingent on the incentive structures within which local discretion is exercised.

Second, our findings offer granular, micro-level evidence for multi-task principal–agent theory. Holmstrom and Milgrom (23) demonstrated that when agents face multiple tasks varying in measurability, they systematically allocate effort toward the more measurable (24, 25). Applied to our context, hospital construction generates quantifiable outputs that fit cadre evaluation templates, whereas older adult care beds yield diffuse outcomes harder to capture in annual performance metrics. Our quantitative finding that fiscal self-sufficiency exacerbates this imbalance, combined with qualitative evidence that officials distinguish between “landmark” and “routine” spending, provides a vivid empirical instantiation of this mechanism in a non-Western institutional setting.

Third, our study extends scholarship on the “medical–social care divide” in comparative welfare state research. Researchers have long noted that health systems command greater political priority and fiscal resources than long-term care systems across OECD countries (26, 27). Our study demonstrates that this divide is not merely a feature of Western welfare regimes or a legacy of historical path dependence. It can be actively reproduced, and even amplified, by contemporary fiscal governance structures—specifically, by the interaction between decentralized fiscal autonomy and visibility-biased performance incentives (28).

Beyond its engagement with these three literatures, the concept of uncoordinated fiscal autonomy carries implications for two broader theoretical debates. The first concerns the relationship between fiscal capacity and governance capacity within the “state capacity” literature (29). A core proposition in this tradition holds that fiscal capacity is a necessary precondition for effective public goods provision. Our findings do not contradict this at the aggregate level; rather, they reveal that the composition of public spending—not merely its magnitude—determines whether fiscal capacity translates into coordinated service delivery. Fiscal capacity, absent complementary “allocative governance capacity”—the institutional mechanisms that ensure expenditure matches multidimensional social needs—can produce perverse distributional outcomes (15).

The second concerns the structure of bureaucratic accountability. The fiscal paradox we identify is, at its root, a problem of misaligned incentives within a system of vertical (upward) accountability (30). County officials in China are evaluated primarily by higher levels of government, and the metrics that dominate these evaluations reward precisely the type of lopsided investment we document (31). In systems with stronger horizontal accountability to service users, one might expect unmet demand for older adult care to generate countervailing political pressure. The fact that the aging rate itself operates as a driver of coordination (H1) suggests that such pressure exists but requires a threshold intensity to overcome bureaucratic inertia. Uncoordinated fiscal autonomy thus captures a specific configuration: vertically accountable local governments, equipped with discretionary fiscal resources, operating under performance metrics that valorize visibility over balance. This configuration is not unique to China—it resonates with dynamics in other centralized or quasi-federal systems where subnational officials face career incentives tied to measurable outputs rather than to the multidimensional welfare of aging populations. That this pattern emerges even in Zhejiang—one of China’s most fiscally robust provinces—suggests that the fiscal paradox is unlikely to be an artifact of resource scarcity; rather, it may be more pronounced in fiscally strained inland regions, where the tension between visible medical investments and less conspicuous older adult care provision is structurally sharper.

These theoretical extensions transform uncoordinated fiscal autonomy from a descriptive label into a diagnostic concept. It identifies the institutional preconditions under which fiscal abundance becomes a liability for achieving structural coordination between medical and older adult care resources, and it specifies two structural remedies: conditional intergovernmental transfers (to impose allocative governance) and reformed performance evaluation (to realign vertical accountability with multidimensional outcomes).

### 4.3 Addressing alternative explanations

The interpretation advanced above must be weighed against plausible alternative explanations.

Differential medical need. One might argue that fiscally strong counties exhibit lower CCD not because of biased investment, but because they face genuinely higher demand for acute medical services—as regional medical hubs serving patients from neighboring counties. Two considerations weigh against this interpretation. First, our models control for the aging rate and urbanization rate, which should absorb a substantial portion of cross-county variation in medical demand. Second, our qualitative interviews in high-fiscal-capacity counties did not reveal narratives of unmet medical need driving hospital expansion; rather, officials described expansion in terms of political salience and landmark achievement.

Reverse causality. Could counties with low CCD—because they have over-invested in medical infrastructure—subsequently experience faster economic growth, which in turn raises their fiscal self-sufficiency? This reverse-causality narrative would invert the direction of our proposed mechanism. Our short panel limits formal tests of this hypothesis, but the economic returns to hospital investment are typically long-term and diffuse, unlikely to manifest within a three-year window. Moreover, our qualitative evidence consistently described fiscal self-sufficiency as a pre-existing structural condition that shaped budget priorities, not as a consequence of those priorities.

Omitted variable bias. A third possibility is that some unobserved county characteristic jointly determines both fiscal self-sufficiency and CCD. Our two-way fixed effects specification already controls for time-invariant county heterogeneity, absorbing any stable omitted factor such as historical legacy or persistent geographic features. Time-varying omitted factors remain a concern, but the qualitative component provides process-tracing evidence that visibility-biased expenditure preferences were actively at work in the budget formulation processes we studied. This triangulation between quantitative patterns and qualitative mechanisms strengthens the plausibility of our interpretation.

Measurement artifact. Finally, one might question whether the negative fiscal–CCD association is an artifact of the CCD formula. If fiscally strong counties have substantially more physicians and nurses per capita (inflating *U*₁), this could mechanically depress CCD when *U*₂ does not increase proportionally. However, this is precisely the substantive phenomenon we seek to capture: the medical subsystem has expanded without corresponding development of older adult care infrastructure. The CCD formula does not create this imbalance; it measures it. The sensitivity check using entropy-weighted CCD (Supplementary Table S6) produced consistent results, further reducing concerns about weighting or aggregation artifacts.

In summary, while none of these alternatives can be definitively eliminated in an observational study, the combination of quantitative robustness, qualitative mechanism evidence, and theoretical coherence suggests that the fiscal paradox we identify is a substantive phenomenon warranting serious attention.

### 4.4 Policy implications

Our findings carry four concrete policy implications, each directly traceable to the mechanisms identified above.

First, the design of intergovernmental transfers may need to incorporate mandatory coupling indicators. The association between transfer dependence and higher coordination observed in our qualitative data is consistent with the interpretation that conditional funding may promote balanced allocation, though this mechanism was not directly tested with transfer data in our quantitative analysis. Should future research with direct measures of transfer conditionality confirm our interpretation, central and provincial governments could consider piloting specific, auditable balance requirements into special-purpose transfers for health and older adult care. For instance, matching ratios could be explicitly linked to provided evidence consistent with progress in both subsystems, rather than to aggregate spending or facility counts in either sector alone. Fiscal transfers can improve public service delivery through expenditure restructuring, as evidenced by county-level data across China (14).

Second, performance evaluation systems for county-level officials could be reformed to correct visibility biases. Our finding that fiscally autonomous counties over-invest in high-visibility medical infrastructure reflects a deeper misalignment between what cadre evaluation systems measure and what integrated care requires. Public service investment, unlike economic performance, is negatively associated with cadre promotion—a pattern confirmed by a meta-analysis of 67 studies (31). We suggest that incorporating “cross-sectoral coupling indicators”—specifically, ratios that compare the growth rates or per capita levels of medical and older adult care resources—into the performance scorecards of county governments. This would transform older adult care beds from a “low-visibility” residual category into a tracked, rewarded component of local government performance, thereby recalibrating the incentives that currently drive disproportionate investment toward hospital-centric projects.

Third, the positive effect of demographic pressure should be strategically leveraged. Our finding that aging rates are positively associated with CCD—and the qualitative evidence suggesting a threshold dynamic—implies that counties approaching or exceeding an aging rate of approximately 23% represent a window of political feasibility for integrated care reforms. Policy-makers at provincial and national levels can use demographic projections to identify counties nearing this threshold and proactively deploy technical assistance, pilot funding, and regulatory mandates before service deficits escalate into public dissatisfaction.

Fourth, resource planning denominators could be realigned with actual demand. Our study’s reliance on registered (hukou) population—dictated by current data availability—reflects a broader planning disjuncture in regions of net in-migration. Policy-makers could accelerate the transition from registered-population-based to resident-population-based resource allocation formulas, accompanied by fiscal transfer mechanisms that compensate counties with large in-migrant populations for the resulting increase in service obligations. Without this shift, the mismatch between allocated resources and actual demand will continue to erode the very coordination our CCD measures attempt to capture.

While this study has focused on the structural coordination of healthcare and older adult care resources, we recognize that addressing population aging requires a far broader policy agenda including post-acute care transitions and community-based service models (37). Effective responses to demographic aging extend beyond the health–long-term care interface to encompass multidimensional legislative and institutional reforms. These include adapting labor market regulations to facilitate extended working lives, reforming pension systems to ensure fiscal sustainability while protecting adequacy, developing telecare and digital health infrastructure to support aging in place, and strengthening legal instruments that protect older adults against exclusion and discrimination (32, 33). The structural coordination we examine here should be understood as one critical component within this larger policy ecosystem. Future research could usefully examine how the fiscal paradox we identify—where unconstrained fiscal autonomy produces lopsided investment patterns—manifests across other domains of aging policy, such as the balance between pension generosity and long-term care funding, or between institutional care and community-based services.

### 4.5 Limitations

Several limitations qualify our findings and indicate directions for future research.

First, the use of municipal-level aging rates as county proxies—necessitated by data availability—may mask within-prefecture heterogeneity. Mountainous or rural counties within a municipality likely have higher effective aging rates than the urban core, implying that our estimates may be biased conservatively (toward the null) for these units. To assess whether this measurement limitation drives our results, we re-estimated the main model without the aging variable. The fiscal self-sufficiency coefficient remained negative and marginally significant (*β* = −0.0013, *p* = 0.052; Supplementary Table S4), confirming that the fiscal paradox is robust to the exclusion of the aging proxy. Future studies with access to county-level resident-based aging data should validate our findings.

Second, the short panel period (*T* = 3) precludes analysis of lagged effects and long-term dynamics. The 2022–2024 window captures an immediate post-pandemic adjustment period, which is valuable for establishing baseline evidence for China’s 2025–2027 integrated care action plan, but longer series are needed to distinguish persistent trends from transitory shocks.

Third, our main analysis uses registered (hukou) population as the denominator for all per-capita indicators, consistent with China’s resource planning framework but potentially misaligned with actual service demand in areas of net migration. To assess the sensitivity of our results to this choice, we recalculated the CCD and all subsystem indicators using resident population and re-estimated the main model. The fiscal self-sufficiency coefficient remained negative and statistically significant (*β* = −0.0026, *p* = 0.021; Supplementary Table S5), indicating that the fiscal paradox is robust to the population denominator. Future studies should nonetheless prioritize resident-population-based indicators, particularly in regions with large migrant populations.

Fourth, the study area is limited to three coastal prefectures in Zhejiang. While the region’s economic heterogeneity provides useful variation, generalizing the fiscal paradox to inland provinces, where both fiscal structures and demographic profiles differ substantially (34), requires separate empirical verification.

Fifth, our panel comprises 31 county-level units observed over 3 years (*N* = 93). While the two-way fixed effects specification controls for unobserved time-invariant heterogeneity and common time trends, the small number of clusters and short time dimension limit statistical power and preclude analysis of lagged effects. The negative adjusted *R^2^* in the main model reflects the large number of fixed effects relative to sample size and indicates that the model identifies partial associations rather than comprehensive determinants. Future studies with longer panels and larger samples are needed to validate the patterns observed here.

Sixth, the qualitative component is based on 12 interviews conducted retrospectively in 2026, recalling budget processes from 2022 to 2024. The sample size, while sufficient to reach thematic saturation for the identified mechanisms, was not designed for statistical generalizability. The qualitative findings should therefore be interpreted as providing plausible, context-specific explanations for the quantitative patterns, rather than as definitive proof of causal mechanisms. Future research with larger, prospective qualitative samples or survey-based mechanism measurements could strengthen the evidential basis for the ‘uncoordinated fiscal autonomy’ concept.

Finally, our 31 county-level units comprise three distinct administrative types: urban districts, county-level cities, and rural counties. These types differ in fiscal autonomy, resource allocation authority, and administrative responsibilities. Urban districts, for example, are more directly integrated into municipal fiscal systems and may have less discretionary control over budget allocation than county-level cities. Conversely, rural counties may face greater structural constraints in attracting and retaining healthcare professionals. While our two-way fixed effects specification controls for time-invariant heterogeneity across units—thereby absorbing any stable differences between administrative types—the small sample size precludes separate analyses by type. Future studies with larger samples should examine whether the fiscal paradox varies systematically across these administrative categories.

## 5 Conclusion

This study examined whether greater local fiscal capacity facilitates the structural coordination of healthcare and older adult care resources under population aging. Drawing on a three-year panel of 31 counties in coastal Zhejiang and complementary qualitative interviews, our answer is a qualified “no.” The coupling coordination degree across the study area remained low (mean CCD = 0.419), with no significant improvement over the 2022–2024 period. More importantly, we identified a robust negative within-county association between fiscal self-sufficiency and CCD: periods of higher fiscal autonomy within a county were accompanied by declining structural coordination between medical and older adult care resources.

This finding gives rise to what we term the fiscal paradox of structural coordination—the condition under which fiscal abundance, rather than enabling balanced service provision, produces lopsided investment patterns that undermine cross-sectoral coordination. Through the concept of uncoordinated fiscal autonomy, we propose that this paradox is driven by the interaction between unconstrained fiscal discretion and career incentives that privilege high-visibility medical infrastructure over less politically rewarding older adult care services. By contrast, periods of greater reliance on conditional transfers were associated with more balanced allocation, suggesting that transfer conditions may impose the structural coordination that autonomous periods fail to self-generate. These findings challenge the implicit assumption in fiscal federalism theory that decentralized fiscal capacity inherently promotes alignment with local needs, and suggest that institutional conditionality may be a necessary complement to fiscal devolution for achieving cross-sectoral coordination.

Our findings, combined with qualitative evidence, generate three testable hypotheses for future research: (1) embedding coupling indicators into intergovernmental transfers may promote balanced resource allocation; (2) reforming performance evaluation systems to incorporate cross-sectoral balance metrics could correct visibility biases; and (3) demographic projections could be used proactively to identify counties approaching the threshold where aging pressure creates political feasibility for coordination reforms. Each proposition merits empirical validation through policy pilots and impact evaluations.

These conclusions are qualified by several limitations, including the use of municipal-level aging proxies, the short panel period, and the geographic confinement to three coastal prefectures. Whether the fiscal paradox generalizes to inland provinces with different fiscal structures and demographic profiles remains an open empirical question. Extending our analytical framework to longer time series, incorporating resident-based aging data, and conducting comparative studies across fiscally contrasting regions represent natural next steps.

If confirmed in broader settings, our core insight carries implications beyond China. In other rapidly aging middle-income countries where subnational governments possess fiscal discretion but operate under accountability structures that privilege measurable, facility-based outputs over long-term care services, analogous dynamics may emerge. Ensuring that fiscal capacity translates into structurally coordinated care—rather than into well-funded but disjointed subsystems—may prove to be one of the defining governance challenges of the coming decades.

## Data Availability

The original contributions presented in the study are included in the article/Supplementary material, further inquiries can be directed to the corresponding author.
