# Supplementary material for: Does wealth hinder structural coordination? Fiscal capacity and the coordination of healthcare and older adult care in China
Source: Front Public Health. 2026 Jul 29;14:1892675. doi: 10.3389/fpubh.2026.1892675 (PMC13463411; doi:10.3389/fpubh.2026.1892675)
Supplement: Supplementary file 1 [file Table_2.DOCX]

**Supplementary Table S1**. Descriptive statistics by year (2022–2024).

| **Variable** | **Year** | **Mean** | **SD** | **Min** | **Max** |
| --- | --- | --- | --- | --- | --- |
| **Beds per 1,000** | 2022 | 6.48 | 3.88 | 2.12 | 19.03 |
|  | 2023 | 6.88 | 4.07 | 2.36 | 19.02 |
|  | 2024 | 6.88 | 4.24 | 1.82 | 20.20 |
| **Physicians per 1,000** | 2022 | 4.67 | 2.34 | 2.34 | 10.75 |
|  | 2023 | 4.89 | 2.43 | 2.44 | 11.08 |
|  | 2024 | 5.12 | 2.48 | 2.58 | 11.44 |
| **Nurses per 1,000** | 2022 | 4.85 | 2.90 | 1.98 | 12.71 |
|  | 2023 | 5.18 | 3.00 | 2.23 | 13.30 |
|  | 2024 | 5.28 | 3.01 | 2.25 | 13.57 |
| **Elderly care beds per 1,000** | 2022 | 5.13 | 3.11 | 1.77 | 13.97 |
|  | 2023 | 4.74 | 2.70 | 1.66 | 11.82 |
|  | 2024 | 5.15 | 3.49 | 1.65 | 18.96 |
| **GDP per capita (RMB)** | 2022 | 128,553 | 61,870 | 56,666 | 337,602 |
|  | 2023 | 120,080 | 56,183 | 53,277 | 307,860 |
|  | 2024 | 114,370 | 55,771 | 49,171 | 304,319 |
| **Fiscal self-sufficiency rate (%)** | 2022 | 62.22 | 25.69 | 20.14 | 109.99 |
|  | 2023 | 64.50 | 25.48 | 21.25 | 116.14 |
|  | 2024 | 60.67 | 24.25 | 21.29 | 114.07 |
| **Aging rate (%)** | 2022 | 26.28 | 2.99 | 23.15 | 30.15 |
|  | 2023 | 25.07 | 2.97 | 22.03 | 28.97 |
|  | 2024 | 23.55 | 2.93 | 20.58 | 27.42 |
| **Urbanization rate (%)** | 2022 | 73.15 | 13.80 | 50.30 | 99.90 |
|  | 2023 | 71.66 | 14.39 | 47.70 | 99.90 |
|  | 2024 | 70.63 | 14.72 | 46.12 | 99.92 |
| **Coupling coordination degree (CCD)** | 2022 | 0.413 | 0.16 | 0.11 | 0.69 |
|  | 2023 | 0.415 | 0.17 | 0.09 | 0.70 |
|  | 2024 | 0.430 | 0.17 | 0.00 | 0.68 |

**Note:** CCD = 0.000 occurred for one county-year observation (Yongjia, 2024). GDP per capita in original RMB yuan. Each year N = 31 (total N = 93).

**Supplementary Table S2**. Robustness check: regression results excluding the observation with CCD = 0 (Yongjia, 2024).

| **Variable** | **Coefficient (Cluster-robust SE)** |
| --- | --- |
| Aging rate (%) | 0.090*(0.0417) |
| Log GDP per capita | 0.127(0.1297) |
| Fiscal self-sufficiency rate (%) | -0.00144*(0.000628) |
| Urbanization rate (%) | 0.000062(0.000823) |
| Observations | 92 |
| R² (within) | 0.129 |
| Adjusted R² | −0.442 |
| Model F-statistic (df=4,55) | 2.03 |

**Note**: Standard errors clustered at the county level are shown in parentheses. The model includes county and year fixed effects. The observation with CCD = 0 (Yongjia county, 2024) was excluded. * p < 0.05.

**Supplementary Table S3**. Robustness check: alternative panel estimators (dependent variable: CCD).

| **Variable** | **Pooled OLS** | **Random Effects** | **Fixed Effects** |
| --- | --- | --- | --- |
| Aging rate | 0.0181*** (0.0060) | 0.0296*** (0.0101) | 0.1281** (0.0565) |
| Log GDP per capita | 0.0787 (0.0536) | 0.1439** (0.0682) | 0.1800 (0.1708) |
| Fiscal self-sufficiency | 0.0009 (0.0008) | –0.0010 (0.0006) | –0.0018** (0.0007) |
| Urbanization rate | 0.0030*** (0.0011) | 0.0009 (0.0009) | 0.0001 (0.0006) |
| Constant | –1.2156** (0.5122) | –1.9515*** (0.6555) |  |
| County fixed effects | No | No | Yes |
| Year fixed effects | Yes | Yes | Yes |
| Clustered SE (county) | No | No | Yes |
| Observations | 93 | 93 | 93 |
| R² | 0.5620 | 0.3295 | 0.2465 |
| Adjusted R² | 0.5421 | 0.2828 | –0.2379 |

**Note**: Standard errors in parentheses. County-clustered robust standard errors for the fixed effects model. **p* < 0.1; ***p* < 0.05; ****p* < 0.01. Each year N = 31 (total N = 93)

**Supplementary Table S4**. Sensitivity analysis excluding the ageing rate (two‑way fixed effects, dependent variable: CCD).

| **Variable** | **Coefficient** | **Cluster‑robust SE** | ***t*‑value** | ***p*‑value** |
| --- | --- | --- | --- | --- |
| Log GDP per capita | 0.0538 | 0.2093 | 0.26 | 0.798 |
| Fiscal self‑sufficiency | −0.0013* | 0.00066 | −1.98 | 0.052 |
| Urbanization rate | 0.0000 | 0.0006 | 0.00 | 0.999 |
| Observations | 93 |  |  |  |
| R² (within) | 0.063 |  |  |  |
| Adjusted R² | −0.513 |  |  |  |
| County FE | Yes |  |  |  |
| Year FE | Yes |  |  |  |

**Notes:** Cluster‑robust standard errors (county level) in parentheses. * *p* < 0.1. The fiscal self‑sufficiency coefficient remains negative and marginally significant, confirming that the main finding is robust to exclusion of the ageing proxy.

**Supplementary Table S5.** Sensitivity analysis using resident population as denominator (two-way fixed effects, dependent variable: CCD).

| **Variable** | **Coefficient** | **Cluster‑robust SE** | ***t*‑value** | ***p*‑value** |
| --- | --- | --- | --- | --- |
| Aging rate | 0.1235* | 0.0481 | 2.57 | 0.013 |
| Log GDP per capita | 0.2018 | 0.1917 | 1.05 | 0.297 |
| **Fiscal self‑sufficiency** | **−0.0026*** | **0.0011** | **−2.37** | **0.021** |
| Urbanization rate | −0.0001 | 0.0007 | −0.17 | 0.862 |
| Observations | 93 |  |  |  |
| R² (within) | 0.183 |  |  |  |
| Adjusted R² | −0.342 |  |  |  |
| County FE | Yes |  |  |  |
| Year FE | Yes |  |  |  |

**Notes**: All per‑capita indicators are calculated using resident population. Standard errors clustered at the county level. * *p* < 0.05.

**Supplementary Table S6.** Entropy-weighted CCD sensitivity analysis (two-way fixed effects).

| **Variable** | **Coefficient** | **Cluster‑robust SE** | ***t*‑value** | ***p*‑value** |
| --- | --- | --- | --- | --- |
| Aging rate | 0.1204* | 0.0538 | 2.24 | 0.029 |
| Log GDP per capita | 0.1657 | 0.1628 | 1.02 | 0.313 |
| **Fiscal self‑sufficiency** | **−0.0017*** | **0.00068** | **−2.48** | **0.016** |
| Urbanization rate | 0.0001 | 0.0006 | 0.13 | 0.900 |
| Observations | 93 |  |  |  |
| R² (within) | 0.178 |  |  |  |
| Adjusted R² | −0.350 |  |  |  |
| County FE | Yes |  |  |  |
| Year FE | Yes |  |  |  |

**Notes**: Standard errors clustered at the county level. *p* < 0.05. The fiscal self‑sufficiency coefficient remains negative and statistically significant, confirming that the main finding is robust to alternative weighting schemes.

**Supplementary File S7**. Qualitative research transparency document.

1. COREQ (COnsolidated criteria for REporting Qualitative research) Checklist

(References to manuscript section numbers correspond to the main text.)

| **No.** | **Item** | **Guide questions/description** | **Reported on page/section** |
| --- | --- | --- | --- |
| Domain 1: Research team and reflexivity | | | |
| Personal Characteristics | | | |
| 1 | Interviewer/facilitator | Which author(s) conducted the interview or focus group? | Section 2.5 (first author) |
| 2 | Credentials | What were the researcher’s credentials? E.g., PhD, MD | Section 2.5 (postgraduate training in qualitative methods) |
| 3 | Occupation | What was their occupation at the time of the study? | Academic researcher |
| 4 | Gender | Was the researcher male or female? | Male |
| 5 | Experience and training | What experience or training did the researcher have? | Section 2.5 (postgraduate qualitative training; prior experience interviewing public sector professionals in China) |
| Domain 2: Study design | | | |
| Theoretical framework | | | |
| 9 | Methodological orientation and Theory | What methodological orientation was stated to underpin the study? e.g., grounded theory, discourse analysis, ethnography, phenomenology, content analysis | Thematic analysis (Braun & Clarke, 2006); combined deductive and inductive approaches (Section 2.5) |
| Participant selection | | | |
| 10 | Sampling | How were participants selected? e.g., purposive, convenience, consecutive, snowball | Purposive, maximum variation sampling on fiscal self-sufficiency and ageing rate (Section 2.5) |
| 11 | Method of approach | How were participants approached? e.g., face-to-face, telephone, mail, email | Through formal invitation via institutional affiliations |
| 12 | Sample size | How many participants were in the study? | 12 (Section 2.5) |
| 13 | Non-participation | How many people refused to participate or dropped out? Reasons? | None reported; all invited informants agreed to participate |
| Setting |  |  |  |
| 14 | Setting of data collection | Where was the data collected? e.g., home, clinic, workplace | Participants’ workplaces (n=9) or neutral meeting rooms in municipal government service centers (n=3) (Section 2.5) |
| 15 | Presence of non-participants | Was anyone else present besides the participants and researchers? | Only the interviewer and participant were present |
| 16 | Description of sample | What are the important characteristics of the sample? e.g., demographic data, date | County health bureaus (4), civil affairs bureaus (4), finance departments (4); all held positions with direct engagement in budget or service planning (Section 2.5) |
| Data collection | | | |
| 17 | Interview guide | Were questions, prompts, guides provided by the authors? Was it pilot tested? | Yes; semi-structured topic guide based on H1–H3; pilot-tested with 2 informants (Section 2.5 and Appendix 1 of this file) |
| 18 | Repeat interviews | Were repeat interviews carried out? | No; single-session interviews |
| 19 | Audio/visual recording | Did the research use audio or visual recording to collect the data? | Audio recorded with written informed consent (Section 2.5) |
| 20 | Field notes | Were field notes made during and/or after the interview or focus group? | Yes, during and immediately after each interview (Section 2.5) |
| 21 | Duration | What was the duration of the interviews or focus group? | 40–70 minutes (mean 52 minutes) (Section 2.5) |
| 22 | Data saturation | Was data saturation discussed? | Yes; after coding 10 transcripts no new themes emerged; final 2 confirmed saturation (Section 2.5) |
| 23 | Transcripts returned | Were transcripts returned to participants for comment and/or correction? | No |
| Domain 3: Analysis and findings | | | |
| Data analysis | | | |
| 24 | Number of data coders | How many data coders coded the data? | Two (first author and a co-author) (Section 2.5) |
| 25 | Description of the coding tree | Did authors provide a description of the coding tree? | Yes; initial deductive codes from H1–H3 plus emergent inductive codes; coding framework provided in Section 3 of this file |
| 26 | Derivation of themes | Were themes identified in advance or derived from the data? | Both deductive and inductive (Section 2.5) |
| 27 | Software | What software, if applicable, was used to manage the data? | Manual coding without software (Section 2.5) |
| 28 | Participant checking | Did participants provide feedback on the findings? | No |
| Reporting |  |  |  |
| 29 | Quotations presented | Were participant quotations presented to illustrate the themes/findings? Was each quotation identified? e.g., participant number | Yes; quotations are attributed to informant roles (e.g., “health bureau official”, “civil affairs official”) in Section 4.1 |
| 30 | Data and findings consistent | Was there consistency between the data presented and the findings? | Yes |
| 31 | Clarity of major themes | Were major themes clearly presented in the findings? | Yes (demand-pressure, fiscal autonomy bias, transfer conditionality) |
| 32 | Clarity of minor themes | Is there a description of diverse cases or discussion of minor themes? | Yes; e.g., “bureaucratic inertia”, “landmark vs. routine spending” reported |

**2. Interview Topic Guide**

Note: The following questions served as a flexible guide. Probes and follow-up questions were tailored to each informant’s role and responses.

Introduction

Confirm consent and confidentiality

Explain the purpose of the study (understanding how county-level resource allocation shapes medical–elderly care coordination)

Section A: Budget formulation process

(1) Could you walk me through the annual budget process for health and elderly care in your county?

(2) Who are the main actors involved in setting budget priorities? How are competing demands resolved?

(3) What information or indicators are used to justify resource requests for health and elderly care?

Section B: Priorities and trade-offs

(4) In your experience, are there differences in how investments in medical infrastructure (e.g., hospitals, equipment) are prioritized compared to elderly care beds? Why?

(5) Can you describe a recent instance where a choice had to be made between expanding a hospital and expanding elderly care facilities? What drove the final decision?

(6) How would you characterize the political visibility of health projects versus elderly care services?

Section C: Intergovernmental transfers and conditions

(7) What types of intergovernmental transfers does your county receive for medical–elderly care integration?

(8) Do these transfers come with specific conditions or matching requirements? How do they influence your allocation decisions?

(9) How would the county’s resource allocation pattern change if these transfers were not earmarked?

Section D: Local implementation of integrated care policies

(10) How are national integrated medical–elderly care policies translated into concrete actions at the county level?

(11) What are the main obstacles you face in implementing integrated care?

(12) Is there anything else you would like to add regarding the coordination of health and elderly care resources?

**3. Coding Framework (Thematic Map)**

The final coding framework was structured into four overarching themes, derived from the three a priori hypotheses (H1–H3) and augmented by inductively generated categories.

| Theme | Sub-themes | Example codes |
| --- | --- | --- |
| 1. Demographic demand pressure (H1) | Threshold dynamics; bureaucratic inertia; citizen pressure | “aging rate crossed 23%”; “policy slogan became performance target”; “complaints from families”; “unmet needs” |
| 2. Fiscal autonomy & visibility bias (H2) | Landmark vs. routine spending; career incentives; residual status of elderly care | “landmark achievements”; “distinguish our district”; “basic livelihood spending”; “hospital expansion”; “advanced equipment”; “performance evaluations” |
| 3. Transfer conditionality & balanced allocation (H3) | Bundled funding; compliance-driven coordination; unified packages | “bundled package”; “matching funds come bundled”; “neglect one side, lose funding for both”; “provincial requirements” |
| 4. Institutional fragmentation & inertia | Departmental silos; bureaucratic inertia; lack of cross-sectoral coordination | “fragmented responsibilities”; “health bureau vs. civil affairs”; “nobody initiates integration”; “routine, not innovative” |

**4. Data Saturation Documentation**

Saturation was assessed using the approach recommended by Braun and Clarke (2006, 2021), focusing on the emergence of new codes rather than merely reaching informational redundancy.

Coding process: Transcripts were analyzed in batches of two, ordered by data collection date. The initial coding framework (derived from H1–H3) was applied to the first four transcripts; new inductive codes were added as they appeared.

Tracking new codes: The number of new, unique codes generated was recorded for each transcript. After coding the 8th transcript, the rate of new code generation dropped sharply (no new codes in transcripts 9 and 10, and only one minor code in transcript 11 which was merged into an existing sub-theme).

Confirmation: The final two transcripts (11 and 12) were coded independently by the second coder, yielding no additional themes or sub-themes and an intercoder agreement >90%.

Saturation decision: Thematic saturation was considered achieved after the 10th transcript. The last two transcripts served as a confirmatory step.

This documentation confirms that the final thematic structure captures the full range of relevant perspectives from the sampled informants.
